# Lamin variants cause cardiac arrhythmogenicity in *Drosophila*

**DOI:** 10.1242/dmm.052424

**Published:** 2025-07-25

**Authors:** Stan W. van Wijk, Puck Vree, Fabries G. Huiskes, Reinier L. van der Palen, Aiste Liutkute, Niels Voigt, Lori L. Wallrath, Bianca J. J. M. Brundel

**Affiliations:** ^1^ Department of Physiology, Amsterdam University Medical Centers, Vrije Universiteit Amsterdam, Cardiovascular Sciences, Heart Failure and Arrhythmias, 1081 HZ Amsterdam, The Netherlands; ^2^ Institute of Pharmacology and Toxicology, University Medical Center Göttingen, 37075 Göttingen, Germany; ^3^ DZHK (German Center for Cardiovascular Research), Partner Site Lower Saxony, 37075 Göttingen, Germany; ^4^ Cluster of Excellence “Multiscale Bioimaging: From Molecular Machines to Networks of Excitable Cells” (MBExC), University of Göttingen, 37075 Göttingen, Germany; ^5^ Department of Biochemistry and Molecular Biology, University of Iowa, Iowa City, IA 52242, USA

**Keywords:** Atrial fibrillation, DNA damage, *Drosophila melanogaster*, Heart wall, Lamin A/C variants, Microtubules

## Abstract

Atrial fibrillation (AF), the most common progressive cardiac arrhythmia, is associated with serious complications such as stroke and heart failure. Although common risk factors underlie AF onset, in 15% of the affected population, AF may have a genetic cause. Here, we investigated how *LMNA* variants cause cardiac arrhythmicity. *Drosophila melanogaster* strains were generated possessing the analogous variants in the *Drosophila* orthologue of human lamin A/C (*LMNA*), *Lamin C* (*LamC*). Heart wall movements in prepupae were recorded before (BTP) and after (ATP) tachypacing. ATP, flies expressing wild-type *LamC*, and the variants ΔN and p.R205W showed a significant reduction in heart rate (HR), but the arrhythmia index (AI) was not affected, compared to BTP. By contrast, those expressing p.N210K and p.R264Q showed a significant reduction in HR and increased AI, compared to BTP. p.N210K- and p.R264Q-expressing prepupae showed contrasting effects after pharmacological intervention with microtubule stabilizer taxol. Taxol attenuated the arrhythmogenicity in p.N210K-expressing prepupae, but aggravated it in p.R264Q-expressing prepupae. These findings suggest that different lamin variants trigger distinct molecular pathways that drive arrhythmogenic effects in *Drosophila.*

## Teaser

**Summary:** Atrial fibrillation-associated lamin variants trigger distinct molecular pathways that drive arrhythmogenic effects in *Drosophila*.

## INTRODUCTION

Atrial fibrillation (AF) is the most common progressive arrhythmia and is associated with various complications such as stroke and heart failure (Van Gelder et al., 2024). Aging and common risk factors – such as lifestyle-related hypertension, diabetes and obesity – are associated with the onset of ‘wear and tear’ AF (Van Gelder et al., 2024; Bode et al., 2024). However, in 15% of patients with AF, there is a familial link for AF without the presence of underlying risk factors (Brundel et al., 2022). These patients may have an inherited genetic predisposition to develop AF (Darbar et al., 2003; Ellinor et al., 2005; Palatinus and Das, 2015; Tucker et al., 2016). Several gene variants have been identified to be associated with the onset of AF, with a majority of these variants located in genes that encode ion channels or cytoskeletal-associated proteins (Brundel et al., 2022). However, variants in the lamin A/C (*LMNA*) gene encoding nucleoskeletal proteins called lamins also cause AF (Zhang et al., 2021).

Lamins are ubiquitously expressed type V intermediate filament proteins. They possess a conserved protein domain structure consisting of a globular head, coil-coil rod, and tail domain possessing an Ig-like fold (Ahn et al., 2019). Lamins dimerize through the rod domain and interact in a head-to-tail manner to form filaments (Ahn et al., 2019). The filaments interact laterally to form a meshwork that lines the inner nuclear membrane. This meshwork provides structural support for the nucleus and plays a role in organizing the chromatin (Gruenbaum et al., 2003; Aebi et al., 1986; Sapra et al., 2020). In addition, lamins interact with the linker of nucleoskeletal and cytoskeleton (LINC) complex that transmits mechanical force from the cytoplasm to the nucleus (Crisp et al., 2006; Lammerding et al., 2004). Humans possess two types of lamin, A- and B-types, which differ in developmental expression patterns and structural properties. Human A-type lamins, lamin A and C, are alternatively spliced products from the *LMNA* gene. Thus far, 498 identified variants in *LMNA* have been reported and linked to a wide range of diseases (Crasto et al., 2020). Several of these variants are associated with cardiovascular diseases, including the development of AF and dilated cardiomyopathy. Notably, in 50% of individuals carrying *LMNA* variants, the onset of AF precedes the development of dilated cardiomyopathy or other ventricular arrhythmic-related cardiomyopathies by several years (Captur et al., 2018; Kumar et al., 2016; van Berlo et al., 2005), indicating that *LMNA* variants drive AF. How *LMNA* variants contribute to AF is not well understood.

To examine the role of lamin variants in AF, we utilized *Drosophila*. The *Drosophila* orthologue of human *LMNA* is *Lamin C* (*LamC*), which shares 37% amino acid identity and 57% amino acid similarity (Schulze et al., 2009). Moreover, *LamC* plays crucial roles in structural organization of the nuclear envelope, nuclear lamina and nuclear chromatin, indicating highly conserved function between *Drosophila LamC* and human *LMNA* (Schulze et al., 2009). Transgenic *Drosophila* strains have been generated to study the effects of lamin variants on cellular functions (Walker et al., 2023; Piazza and Wessells, 2011; Duffy, 2002). Here, we investigated the impact of four *LamC* variants – ΔN (Schulze et al., 2009), p.R205W (Bhide et al., 2018), p.N210K (Schulze et al., 2009) and p.R264Q (Hinz et al., 2021), resembling the human *LMNA* variant Δexon 1 (van Tintelen et al., 2007), p.R190W (Arbustini et al., 2002), p.N195K (Fatkin et al., 1999) and p.R249Q (Bonne et al., 2000; Raffaele Di Barletta et al., 2000), respectively, on cardiac arrhythmicity and contractile function. Furthermore, pharmacological intervention with the BRD4 inhibitor RVX-208 and the microtubule-stabilizing drug taxol revealed protective as well as detrimental effects, depending on the specific variant. These findings suggest that variant-specific activation of distinct molecular pathways drives arrhythmogenic effects in *Drosophila.*

## RESULTS

### Pathogenic A-type lamin missense variants are conserved between species

**Fig. 1.**
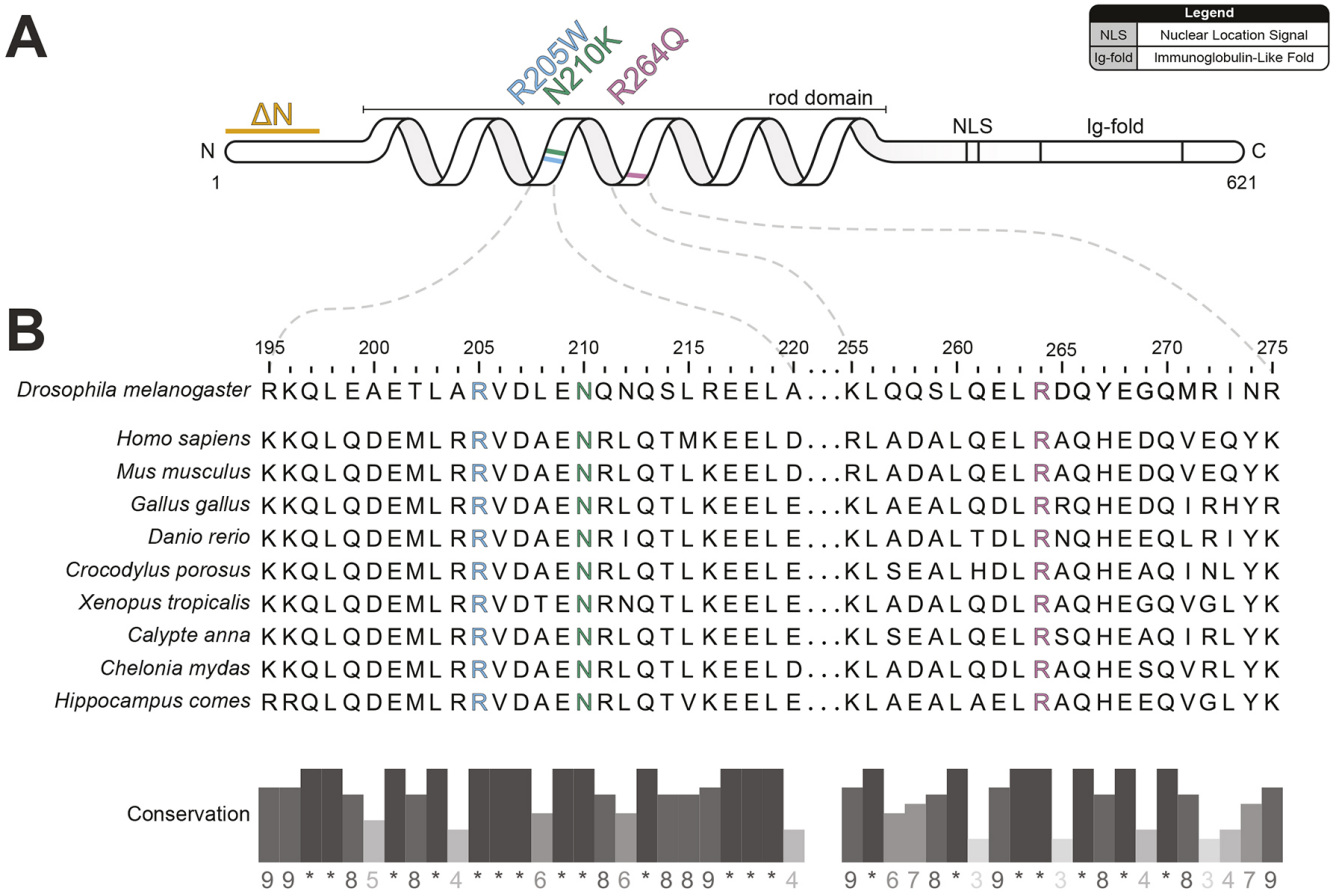
**Protein homology of A-type lamins between species and locations of pathogenic variants.** (A) Schematic of *Drosophila LamC*, with positional highlights corresponding to human *LMNA* pathogenic variants indicated in orange (ΔN), blue (p.R205W), green (p.N210K) and pink (p.R264Q). (B) Alignment of protein sequences of A-type lamins of various species. Numbers indicate only the position of the amino acid in *Drosophila* LamC protein. The conservation of amino acids with pathogenic missense variants suggests a pivotal role of the amino acid in the formation or function of the A-type lamin protein. The positions of the missense variants are highlighted in blue (p.R205W), green (p.N210K) and pink (p.R264Q).

To investigate the arrhythmogenicity of human *LMNA* variants in *Drosophila*, four *Drosophila* strains were generated that express *LamC* variants specifically in the heart (Fig. 1). These variants were selected because they are associated with cardiac disease in humans. *Drosophila LamC* ΔN (Schulze et al., 2009) is comparable to an N-terminal truncated A-type lamin in humans associated with AF (van Tintelen et al., 2007). Additionally, *Drosophila LamC* p.R205W, p.N210K and p.R264Q correspond to pathogenic *LMNA* variants p.R190W (Pethig et al., 2005), p.N195K (Fatkin et al., 1999) and p.R249Q (Hinz et al., 2021; Perepelina et al., 2020), respectively, which are associated with AF. These missense variants affect the rod domain of A-type lamins, which are most associated with muscle laminopathies (Fatkin et al., 1999; Ahn et al., 2019). *LamC* p.R205W and p.N210K are located towards the carboxy end of the 1B coil (Fig. 1A).

### *Drosophila* strains expressing *LamC* variants p.N210K and p.R264Q show increased arrhythmicity after tachypacing

**Fig. 2.**
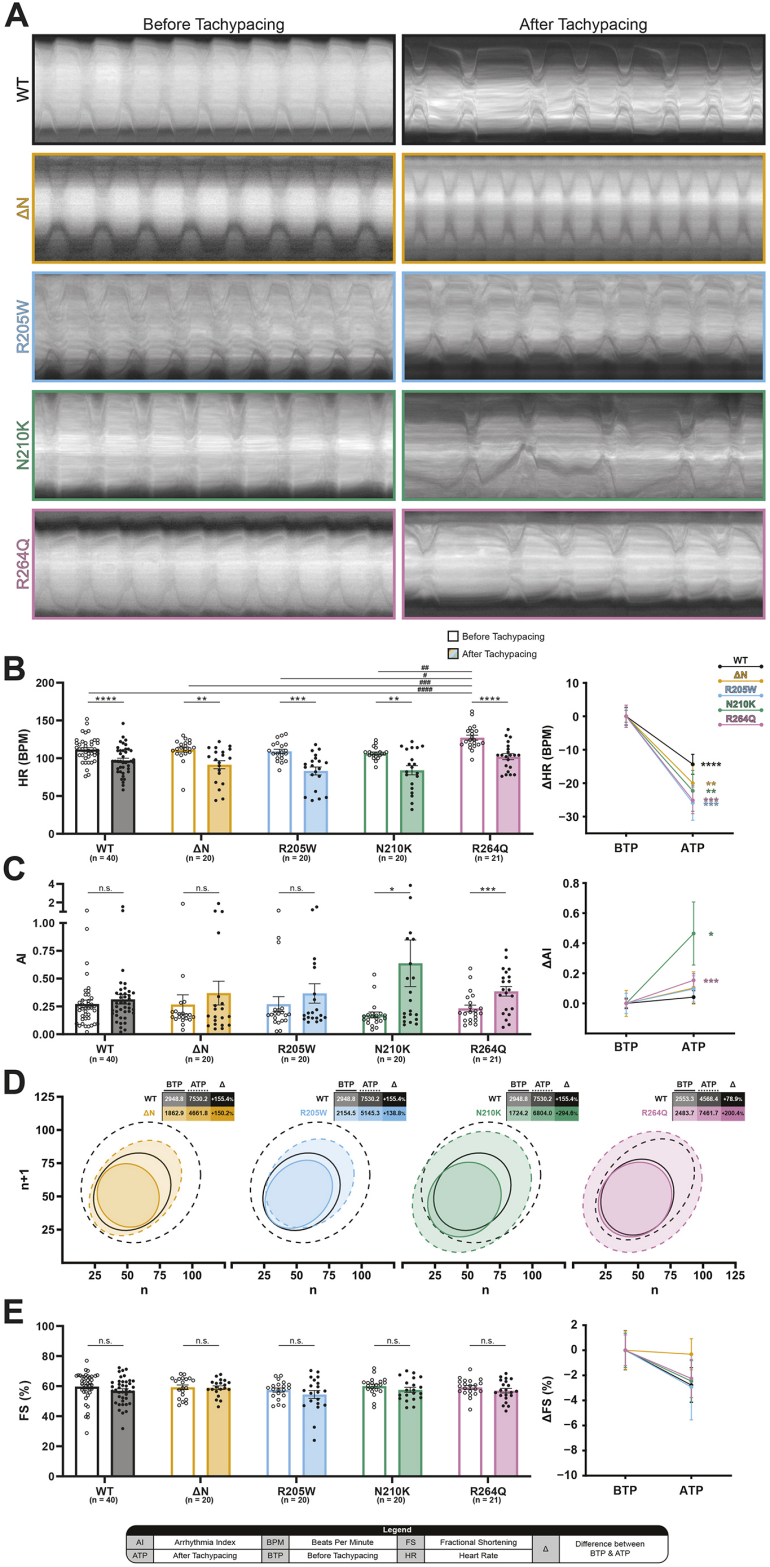
**Prepupae expressing *LamC* p.N210K and p.R264Q display increased AI^ATP^.** (A) Representative kymographs of heart wall contractions of *Drosophila* prepupae expressing wild-type *LamC* (WT; black), and the variants ΔN (orange), p.R205W (blue), p.N210K (green) and p.R264Q (pink), recorded BTP and ATP. (B) Prepupae show a significantly reduced HR^ATP^ in all groups. (C) Strains expressing p.N210K and p.R264Q exhibit a significant increase in AI^ATP^ compared to their respective AI^BTP^. (D) Poincaré plots displaying variability in HR. A 0.99 confidence ellipse describes the point distribution within the graph, with the solid and dashed lines indicating the heart wall contraction variability BTP and ATP, respectively. Areas of the ellipses and the percentile differences (Δ) between BTP and ATP are displayed in the corresponding tables. (E) No changes in FS^ATP^ compared to FS^BTP^ were observed for all strains. The differences between BTP and ATP are also shown in a line graph to highlight the differences between strains (B,C,E, right). All single data points represent a single measurement of a prepupae, bar and line graphs indicate the mean, and error bars represent s.e.m. Pairwise comparisons of means were evaluated with Wilcoxon signed rank test, and comparisons between individual means were evaluated with Kruskal–Wallis test. *N*=2. n.s., not significant; *P<0.05, **P<0.01, ***P<0.001 and ****P<0.0001 compared to the group indicated.

To quantify the impact of *LamC* variants on heart rate (HR) and rhythmicity, *Drosophila* strains carrying wild-type *LamC* (WT) or a variant (ΔN, p.R205W, p.N210K or p.R264Q) were crossed with a cardiac-specific driver strain Hand4.2-Gal4. Using *Drosophila* offers the advantage of visualizing the heart in an intact animal, and, owing to the semi-transparent nature of the pupal case, heart function can be monitored visually (Zhang et al., 2011; Wessells and Bodmer, 2004). The heart wall contractions of F1 prepupae expressing cardiac-specific *LamC* variants were recorded before (BTP) and after (ATP) tachypacing (TP), and summarized in M-mode cardiograms (Fig. 2A). To quantify the impact of TP and *LamC* variant on the function of heart wall contractions, we measured HR^BTP^ and HR^ATP^ (Fig. 2B, left). At baseline, only p.R264Q-expressing prepupae displayed a significantly higher HR^BTP^ compared to that of WT-expressing prepupae, indicating that the heart wall function is affected by this variant. Prepupae expressing the other *LamC* variants showed comparable HR^BTP^ to that of WT-expressing prepupae. TP resulted in a significant decrease in HR in prepupae expressing WT [−14.35±2.99 beats/min (BPM)], ΔN (−20±5.32 BPM), p.R205W (−25.9±5.20 BPM), p.N210K (−22.3±6.10 BPM) and p.R264Q (−25.14±3.98 BPM) (Fig. 2B, right). However, there were no significant differences in HR^ATP^ among the variant-expressing strains, suggesting that all variants are similarly affected by TP.

Next, the arrhythmicity index (AI) was measured BTP and ATP, to quantify the arrhythmogenic effect of the *LamC* variants. The variability in HR is expressed as the AI (Fig. 2C), and the amount of variability is visualized in a Poincaré plot (Fig. 2D). Prepupae expressing the *LamC* variants exhibited no significant changes in AI^BTP^ compared to those expressing WT. AI^ATP^ was comparable to AI^BTP^ in prepupae expressing WT, ΔN and p.R205W. However, prepupae expressing p.N210K [0.46±0.21 arbitrary units (A.U.)] and p.R264Q (0.15±0.04 A.U.) showed a significant increase in AI^ATP^ compared to AI^BTP^ (Fig. 2C,D). These results indicate that there is a *LamC* variant-specific effect on TP-induced cardiac arrhythmicity.

Fractional shortening (FS) was used to quantify the force of contraction of the *Drosophila* heart tube by measuring widest heart wall distances during systole and diastole. There were no significant differences in FS^BTP^ and FS^ATP^ between the different *LamC* variant-expressing strains (Fig. 2E). In short, these findings indicate that all *LamC* variant-expressing strains showed a significant reduction in HR^ATP^, whereas an increase in AI^ATP^ was specifically found in strains expressing p.N210K and p.R264Q, and not in those expressing WT, ΔN and p.R205W, without affecting the FS of the heart tube.

### Pharmacological intervention with RVX-208 and taxol

**Fig. 3.**
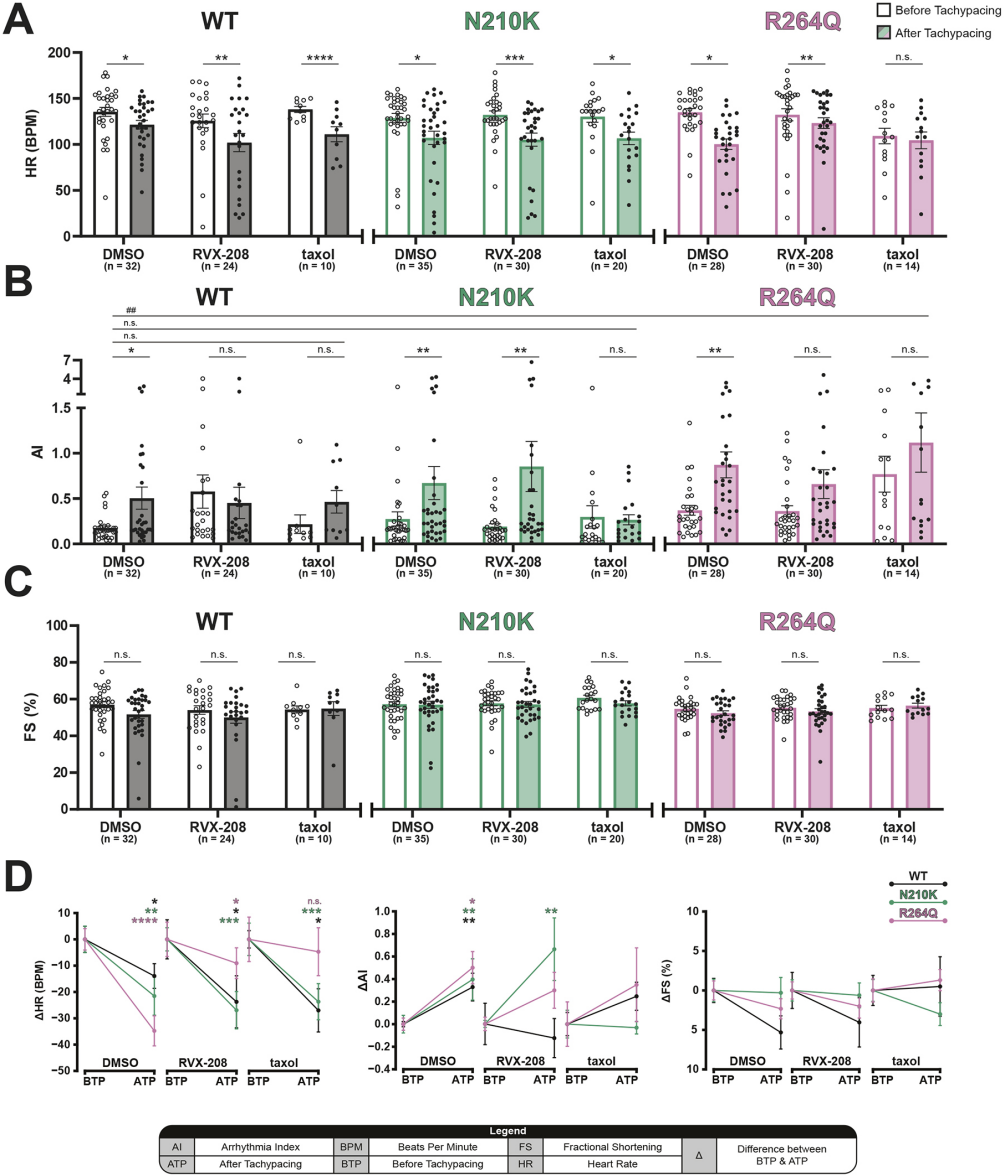
**The effects of BRD4 inhibitor RVX-208 and microtubule stabilizer taxol on AI are *LamC* variant dependent.** Heart wall contractions of *Drosophila* prepupae expressing WT (black), p.N210K (green) and p.R264Q (pink) were recorded BTP and ATP, after exposure to DMSO, RVX-208 or taxol. (A) Tachypacing resulted in a significant reduction in HR^ATP^ compared to the respective HR^BTP^ in most conditions. However, taxol-treated p.R264Q-expressing prepupae showed no significant decrease in HR^ATP^, because p.R264Q-expressing prepupae treated with taxol present a lower HR at baseline compared to the HR^ATP^ of WT-expressing prepupae treated with DMSO. (B) DMSO-treated WT-, p.N210K- and p.R264Q-expressing prepupae all show a significant increase in AI^ATP^. RVX-208 prevents the increase in AI^ATP^ in WT- and p.R264Q-expressing prepupae. Taxol protects against the increase in AI^ATP^ in WT-, p.N210K- and p.R264Q-expressing prepupae. (C) WT-, p.N210K- and p.R264Q-expressing prepupae treated with DMSO, RVX-208 or taxol exhibit no significant difference in FS^ATP^. (D) Line graphs indicating the differences in HR^BTP/ATP^ (left), AI^BTP/ATP^ (middle) and FS^BTP/ATP^ (right) after treatment with DMSO, RVX-208 or taxol. All single data points represent a single measurement of a prepupae, bar and line graphs indicate the mean, and error bars represent s.e.m. Pairwise comparisons of means were evaluated with Wilcoxon signed rank test, and comparisons between individual means were evaluated with Kruskal–Wallis test. *N*=2. n.s., not significant; *P<0.05, **P<0.01, ***P<0.001 and ****P<0.0001 compared to the group indicated.

To elucidate the molecular mechanisms underlying *LamC* p.N210K- and p.R264Q-induced susceptibility to cardiac arrhythmicity, these strains were treated with either the BRD4 inhibitor RVX-208 (apabetalone) or microtubule stabilizer taxol and compared to those treated with vehicle control [dimethyl sulfoxide (DMSO)]. At baseline, RVX-208 and taxol had no effect on HR compared to control except for in p.R264Q-expressing prepupae, which showed reduced HR compared to that of WT-expressing prepupae. TP resulted in a significant reduction in HR^ATP^ in prepupae expressing WT (−13.94±4.69), p.N210K (−21.54±7.25) and p.R264Q (−34.79±5.70) (Fig. 3A,D). This effect could not be rescued by either RVX-208 or taxol.

No effect of RVX-208, taxol or DMSO alone was observed on AI at baseline for all the *LamC*-expressing strains. AI^ATP^ was significantly increased, compared to AI^BTP^, for strains expressing WT, p.N210K and p.R264Q, which was prevented by RVX-208 treatment in those expressing WT and p.R264Q, and taxol treatment in those expressing WT, p.N210K and p.R264Q (Fig. 3B,D). Taxol prevented the increase in AI^ATP^, compared to the AI^BTP^ in WT-expressing prepupae, in WT- and p.N210K-expressing strains. By contrast, taxol increased AI^ATP^, compared to the AI^BTP^ in WT-expressing prepupae, in the p.R264Q strain (Fig. 3B,D).

Finally, the effects of RVX-208 and taxol on FS were studied. No effects of these treatments on FS^BTP/ATP^ for strains expressing WT, p.N210K and p.R264Q were observed, indicating no effect on diastolic and systolic heart wall function (Fig. 2E, Fig. 3C,D).

### Molecular mechanism of pharmacological intervention in *LamC* variants

**Fig. 4.**
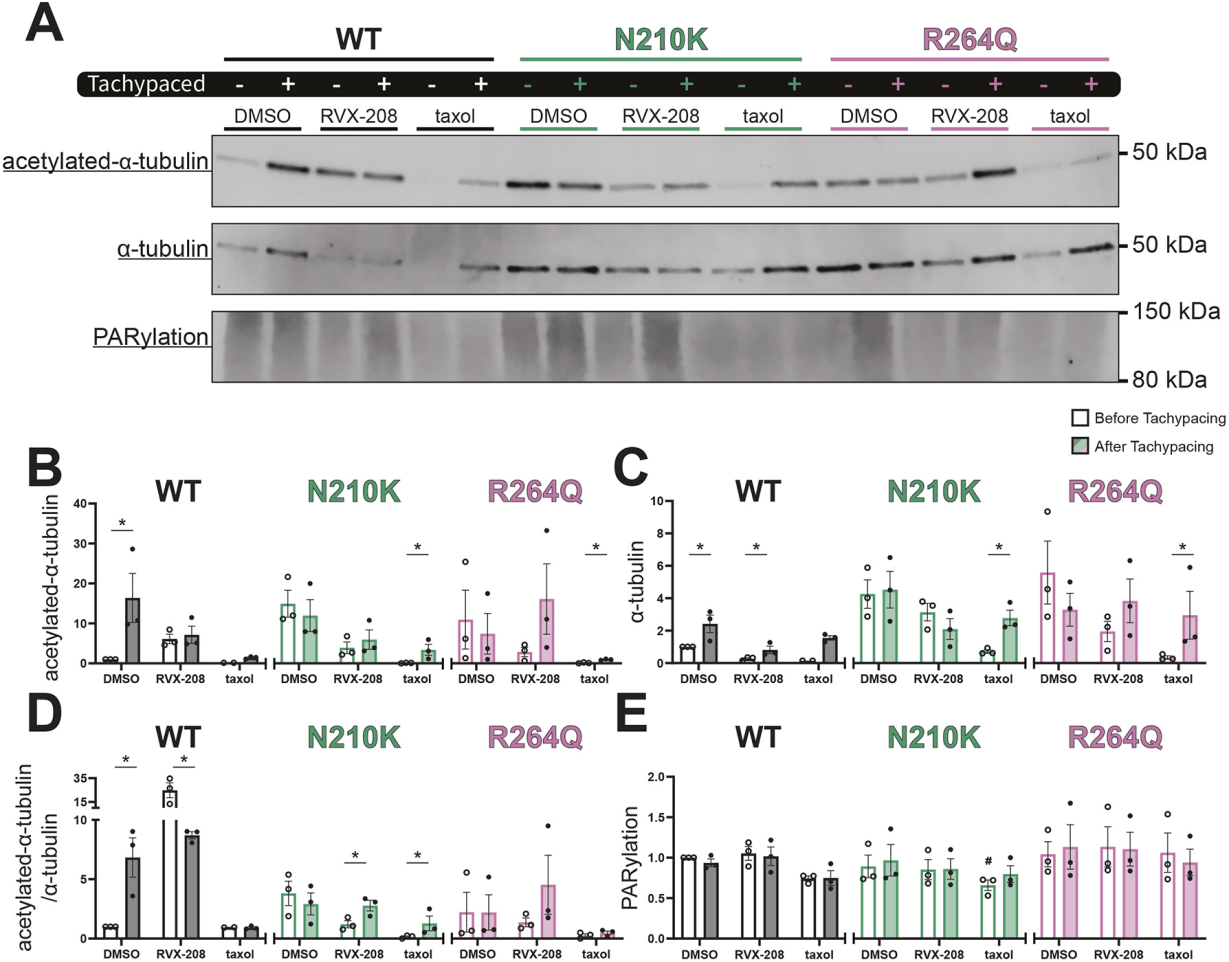
***LamC* variant-expressing prepupae show increased acetylation of α-tubulin after RVX-208 treatment.** (A) Representative western blot analyses of acetylated-α-tubulin, α-tubulin and poly(ADP-ribosyl)ation (PARylation) levels in *Drosophila* prepupae treated with DMSO, RVX-208 or taxol BTP and ATP. (B-E) Quantified data of western blots normalized to the total protein stain (Fig. S1) and displayed as the total amount of acetylated-α-tubulin (B), α-tubulin (C), acetylated-α-tubulin as a ratio of total α-tubulin (D) and PARylation (E) present in whole *Drosophila* prepupae lysates. All single data points represent an individual western blot, bar graphs indicate the mean, and error bars represent s.e.m. Comparisons of means were evaluated with Kruskal–Wallis test. *N*=3. n.s., not significant; *P<0.05 compared to the group indicated.

To further investigate the molecular mechanisms underlying the differences in heart wall contraction and arrhythmogenicity observed in *LamC* variants, markers for DNA damage and the microtubule network were analyzed using western blot analysis of protein extracts from prepupae. Previous studies have linked DNA damage and microtubule network dysfunction with both experimental and clinical AF (Zhang et al., 2014). Therefore, poly(ADP-ribosyl)ation (PARylation), an indicator of genotoxic stress, along with acetylated-α-tubulin, total α-tubulin and their ratio, were quantified (Fig. 4A).

Upon DMSO treatment, the WT-expressing strain demonstrated a significant increase in acetylated-α-tubulin (Fig. 4B), total α-tubulin (Fig. 4C) and the acetylated-α-tubulin/α-tubulin ratio (Fig. 4D) under ATP conditions. By contrast, strains expressing *LamC* variants p.N210K and p.R264Q exhibited no significant changes in α-tubulin levels under these conditions (Fig. 4B-D). Treatment with RVX-208 induced a significant decrease in the acetylated-α-tubulin/α-tubulin ratio in prepupae expressing WT, whereas those expressing p.N210K showed a significant increase in this ratio. Interestingly, taxol treatment also significantly increased the acetylated-α-tubulin/α-tubulin ratio in prepupae expressing p.N210K, like in those expressing RVX-208. In addition, taxol significantly elevated both acetylated-α-tubulin (Fig. 4B) and total α-tubulin (Fig. 4C) expression in prepupae expressing p.N210K. For prepupae expressing p.R264Q, taxol similarly increased acetylated-α-tubulin and total α-tubulin expression; however, the acetylated-α-tubulin/α-tubulin ratio remained unchanged. PARylation levels were comparable across all groups (Fig. 4E).

In short, prepupae expressing WT exhibited increased microtubule acetylation under control conditions (DMSO treatment), whereas this effect was absent in prepupae expressing p.N210K and p.R264Q. RVX-208 treatment had contrasting effects on the WT- and p.N210K-expressing strains, whereas taxol significantly enhanced acetylated-α-tubulin expression in both p.N210K- and p.R264Q-expressing strains. These findings highlight distinct molecular responses of *LamC* variants to microtubule-targeting interventions.

## DISCUSSION

Several families with AF have been identified as carrying variants in the *LMNA* gene, implicating it in AF pathogenesis. In this study, we leveraged *Drosophila* as a validated experimental model for AF (Kervadec et al., 2023; Zhang et al., 2011, 2014, 2019; Li et al., 2022; Wiersma et al., 2017) to explore the mechanisms associated with *LMNA* variants. Our results demonstrate distinct effects of these variants on HR and AI in *Drosophila* prepupae, measured BTP and ATP. We observed that the prepupae expressing WT, ΔN and p.R205W exhibited a reduction in HR^ATP^, compared to HR^BTP^, whereas AI^ATP^ remained unchanged compared to AI^BTP^. In contrast, p.N210K- and p.R264Q-expressing prepupae showed a reduction in HR^ATP^, compared to HR^BTP^, and an increase in AI^ATP^, compared to AI^BTP^, highlighting their greater arrhythmogenic potential. Interestingly, FS was comparable across all strains, suggesting that the structural function of the heart was preserved, despite TP. Pharmacological interventions with the BRD4 inhibitor RVX-208 and the microtubule stabilizer taxol provided further mechanistic insights. RVX-208 prevented reduction in HR^ATP^, particularly in the prepupae expressing the p.R264Q variant. Taxol attenuated the arrhythmogenic effects in the prepupae expressing the p.N210K variant, but, in contrast, exacerbated AI^ATP^ in the prepupae expressing the p.R264Q variant.

In summary, our findings suggest that *LamC* variants, specifically p.N210K and p.R264Q, lead to distinct impairment of heart wall function, characterized by reduced HR and increased arrhythmogenicity. The differential response to pharmacological agents further implies that these variants engage distinct molecular pathways, including the microtubule network, that drive AF pathogenesis. This study suggests the importance of variant-specific therapeutic strategies.

### *LamC* variants and their effects on heart wall function

*LMNA* is a critical component of the nuclear lamina, where it forms a meshwork that lines the inner side of the nuclear envelope (Dittmer and Misteli, 2011). The lamina plays key roles in maintaining nuclear architecture, regulating gene expression and connecting to the LINC complex (Crisp et al., 2006; Lammerding et al., 2004; Wong et al., 2021; Lammerding et al., 2006). The current study shows that *LamC* variants p.N210K (analogous to *LMNA* p.N195K) and p.R264Q (analogous to *LMNA* p.R249Q) result in impairment of heart wall function, reflected by reduced HR and increase in arrhythmogenic events. *LamC* p.N210K and p.R264Q may lead to an altered lamin meshwork and/or nuclear shape (Schulze et al., 2009; Hinz et al., 2021). p.N210K is located in coil 1B of *LamC*, and *LMNA* variants located to this rod domain are known to perturb the polymerization of the lamin meshwork, causing attenuated elasticity of the nuclear lamin and deformed nuclei (Bera et al., 2016). Furthermore, *Lmna^+/−^* mouse embryonic fibroblasts expressing human p.N195K (p.N210K) are less capable of retaining nuclear stiffness after mechanical challenge and show disturbed filament assembly *in vitro* (Zwerger et al., 2013). *LamC* p.R264Q is located in the amino end of coil 2A and causes deformed nuclei in larval body wall muscles (Hinz et al., 2021). Consistent with this, the human equivalent, *LMNA* p.R249Q, causes deformed nuclei in induced pluripotent stem cell-derived cardiomyocytes (Wallace et al., 2023). Abnormally formed nuclear meshwork could lead to different interactions between lamins and the LINC complex, which might affect cytoskeletal organization and, as such, drive impairment of the microtubule network, calcium handling and contractile function of the atrial cardiomyocytes. Such changes have been observed in experimental AF models (Zhang et al., 2014; Yeh et al., 2008) and clinical cases of AF (Ke et al., 2008; Zhang et al., 2014).

These findings are in line with previous studies that showed that *LMNA* variants can affect cardiac tissue, owing to the function of *LMNA* in maintaining structural and functional integrity and proteostasis in cardiomyocytes subjected to mechanical stress, similar to rapid electrical stimulation observed in AF (Crasto et al., 2020). Our results indicate that mechanisms by which *LMNA* variants may drive AF include genotoxic stress and alteration of the microtubule network, as the BRD4 inhibitor RVX-208 prevented the arrhythmogenic effect of TP in *Drosophila* WT- and p.R264Q-expressing strains, and microtubule stabilizer taxol similarly protected against arrhythmogenicity in WT- and p.N210K-expressing strains. These findings are consistent with studies that show *LMNA* variants result in cardiomyocyte nuclear fragility and altered gene expression due to changes in the chromatin landscape that correlate with DNA damage. Similarly, studies have shown increased DNA damage in experimental and clinical cases of AF (Zhang et al., 2019).

Different *LMNA* variants lead to varying degrees of HR, AI and molecular dysfunction, which explains the heterogeneity in AF presentation among affected individuals (Hoorntje et al., 2017; Perovanovic and Hoffman, 2018). Here, *LamC* variants p.N210K and p.R264Q were shown to significantly reduce HR and increase AI in *Drosophila*, suggesting more severe impact of the variants on heart function compared to ΔN, p.R205W and WT. This could be due to variant-specific effects on signaling pathways that drive arrhythmia. ΔN- and p.R205W-expressing strains showed HR reduction but no significant change in AI^ATP^, compared to AI^BTP^, indicating that not all *LamC* variants equally impact heart wall function.

### Mechanistic interventions with RVX-208 and taxol

*LMNA* encodes lamins, essential intermediate filaments that link various cytoskeletal proteins, such as actin and microtubules, to the nucleus (Wong et al., 2021). Studies have shown that preservation of the microtubule network prevents the development of AF (Li et al., 2022; Zhang et al., 2014; Hu et al., 2019). Moreover, acetylation of the microtubules increases rigidity of the microtubule network and prevents its breakdown. In *Drosophila*, dog and *in vitro* HL-1 cardiomyocyte models of AF, acetylation of microtubules protects against the detrimental effects of TP. It has been well described that *LMNA* variants can lead to modulation of the microtubule network (Chatzifrangkeskou et al., 2023). Interestingly, acetylated-α-tubulin increased in response to TP in WT-expressing prepupae, but this increase was absent in p.N210K- and p.R264Q-expressing prepupae, hinting at a protective effect against *LamC* variant-induced arrhythmogenicity and leading to the assumption that the microtubule stabilizer taxol would have a protective effect against TP-induced arrhythmia in *Drosophila* prepupae. Although there was significant improvement in heart wall function in taxol-treated p.N210K-expressing prepupae ATP, and to a lesser extent in WT-expressing prepupae, there seems to be a toxic effect of taxol on arrhythmicity in p.R264Q-expressing prepupae. This heterogeneity might be explained by increased acetylation of the tubulin network, as there is a significant increase ATP in prepupae expressing p.N210K but not in those expressing WT or p.R264Q.

Interestingly, *Drosophila* expressing *LamC* variants ΔN and p.R205W have loss of microtubule organization around the nucleus (Dialynas et al., 2012; Shaw et al., 2022), but did not show an increase in AI^ATP^. *Drosophila LamC* p.R264Q and mouse *Lmna* p.N195K (p.N210K) retain organization of microtubules around the nucleus (Hinz et al., 2021; Pavlov et al., 2024 preprint), but variant-expressing *Drosophila* prepupae have an increase in AI^ATP^. Uncoupling of the LINC complex with the nuclear lamina in *Lmna* p.N195K-expressing mice eliminates the perinuclear microtubular cage around the nucleus, improves cardiac function, reduces nuclear damage and increases survival (Pavlov et al., 2024 preprint). It could be speculated that a perinuclear microtubule cage is arrhythmogenic in the presence of a disturbed lamin meshwork because of improper filament formation by *LMNA* variants. However, further research is warranted to assess the impact of pharmacological microtubule manipulation as well as the role of the perinuclear microtubule cage in *LMNA* variant-induced arrhythmia.

We also observed that treatment with RVX-208 has varying effects on AI in the *LamC* strains: TP-induced arrhythmicity was alleviated in p.R264Q-expressing prepupae but unaffected in WT- and p.N210K-expressing prepupae. The BRD4 inhibitor RVX-208 is involved in multiple molecular pathways but most interestingly used to prevent detrimental consequences of BRD4 activation after genotoxic stress (Van der Feen et al., 2019). A cardiomyocyte-specific *Lmna*-deprived mouse model exhibited improved cardiac function, and abrogated cardiac arrhythmia and fibrosis formation, after treatment with the pan inhibitor of BET bromodomain protein JQ1 (Auguste et al., 2020). Previous studies showed that DNA damage and excessive DNA damage response are involved in the onset of AF (Zhang et al., 2019). Here, RVX-208 protected against heart wall dysfunction in prepupae expressing *LamC* p.R264Q. As BRD4 is involved in regulating gene expression through chromatin remodeling, the protective effect of this inhibitor suggests that *LMNA* variants influence AF through epigenetic dysregulation of pro-arrhythmic genes. By inhibiting BRD4, it might be possible to restore normal gene expression and reduce AF susceptibility.

Our findings are consistent with the fact that lamins play a plethora of roles and emphasize that missense variants, which alter amino acids, can impact distinct molecular processes yet result in a common phenotype of arrhythmia. Our results indicate that a specific treatment could be beneficial for a particular lamin variant yet be detrimental for another. Further research is warranted to investigate variant-specific responses to drug treatment.

### Translation of findings to clinical AF

Using *Drosophila* as a model for AF research offers several advantages but also has limitations. Although *Drosophila* lacks a four-chambered heart, the basic genetic and molecular pathways that govern cardiac function and rhythm regulation are highly conserved between *Drosophila* and humans. Genes linked to AF, such as those involved in ion channel function and cardiac rhythms, as well as structural proteins, including lamins, have orthologues in *Drosophila*. This makes *Drosophila* a valid model for studying fundamental mechanisms of cardiac function and disease processes such as AF. As *Drosophila* allows for easy genetic interventions specifically in the heart, it has been recognized that *Drosophila* is a powerful model for studying AF-related genes and mechanisms (den Hoed et al., 2013) as well as for drug screening (van Marion et al., 2019; Zhang et al., 2011; Hoogstra-Berends et al., 2012). In fact, several findings with drugs have been translated to dog models of AF (Zhang et al., 2014; Wiersma et al., 2017), and the drugs are currently in clinical trials (van Marion et al., 2020; Pool et al., 2023). As demonstrated in the current study, pharmacological agents such as RVX-208 (a BRD4 inhibitor) and taxol have distinct protective effects in *LamC* variant-expressing *Drosophila*. The arrhythmogenic pathways identified in *Drosophila* are likely to be conserved in humans, but the translation of specific molecular targets or therapeutic interventions will require validation in more complex mammalian systems, including induced pluripotent stem cell-derived atrial cardiomyocytes and transgenic mouse models. Previous studies showed that variants in the intermediate filament protein desmin are also linked to AF (van Wijk et al., 2022; Schrickel et al., 2010; Mavroidis et al., 2020). Moreover, a variety of cytoskeletal and sarcomere-associated proteins are linked to AF (van Wijk et al., 2022). However, *Drosophila* express only two intermediate filament proteins, both of which are nuclear lamins (Dittmer and Misteli, 2011). This limits the model's suitability for studying other types of intermediate filaments or their interactions in the context of AF.

In conclusion, this study provides new insights into the distinct molecular pathways activated by different *LMNA* variants contributing to arrhythmogenesis in *Drosophila*. Our findings underscore the need for personalized therapeutic approaches in managing AF, particularly in patients with underlying genetic causes. Further research is warranted to explore the molecular underpinnings of these variant-specific effects, as well as to assess the clinical utility of pharmacological interventions such as BRD4 inhibitors in the treatment of AF with *LMNA* variants.

## MATERIALS AND METHODS

### *Drosophila* culture and drug treatment

To create cardiac-specific transgenic *LamC* strains, the GAL4-UAS system was used (Brand and Perrimon, 1993; Duffy, 2002). *Drosophila* UAS-LamC ΔN (Schulze et al., 2009), p.R205W (Bhide et al., 2018), p.N210K (Schulze et al., 2009), p.R264Q (Hinz et al., 2021) and WT were crossed with Hand4.2-Gal4 driver strain (Sellin et al., 2006) (kindly provided by Professor Dr Paululat, University of Osnabrück, Osnabrück, Germany). Adult flies were transferred to vials with fly food [5% (w/v) yeast (Mauripan, AB Mauri)], 2% (w/v) agar (Roth, 3810.3), 7,5% (w/v) saccharose, 13.1 nM nipagin (Sigma-Aldrich, H3647) and 0.6% (v/v) propionic acid (Sigma-Aldrich, P1386) dissolved in tap water containing either 250 µM RVX-208 (Van der Feen et al., 2019) (MedChemExpress, HY-16652) or 50 nM taxol (Li et al., 2022) (Sanbio, 10461) or the equivalent amount of vehicle (DMSO; Merck, D2650) and incubated at 25°C. After 4 days, the adult flies were discarded, and F1 offspring prepupae, which consumed the drug or vehicle before entering the prepupa stage, were measured in the following days as described previously (Zhang et al., 2019; Li et al., 2022).

#### *Drosophila* TP and cardiac contractile function assays

*Drosophila* prepupae were collected and subjected to TP for 20 min (5 Hz, 20 V, 5 ms pulse duration) on a 1% agarose gel spanning the electrodes of a four-well C-Dish (GE Healthcare, 17-0554-02) pulsed by an IonOptix C-Pace as previously described (Zhang et al., 2019; Li et al., 2022). Prepupae heart wall contractions were recorded BTP and ATP, utilizing high-speed digital video imaging (mvBlueFox3), creating 30-s movies with 100 frame/s. Recordings were converted to M-mode cardiogram images by drawing a 1-pixel-wide line perpendicularly through the heart walls and generating a kymograph using Fiji (Schindelin et al., 2012) (v1.52p). Generated kymographs were analyzed with the BAR plugin (Ferreira et al., 2015) for Fiji (v1.52p), a horizontal line was drawn perpendicularly through the heart walls in the kymograph, and the smoothened plotted profile was used to calculate time between contractions as described previously (Kurnia et al., 2021). To measure the systolic and diastolic diameters, ten lines were drawn between the heart walls at end-diastole and end-systole, distributed across the entire M-mode cardiogram kymograph in Fiji (v1.52p) to ensure a representative average of the whole recording. Contractile dysfunction is defined as a significant reduction in HR, increase in AI or reduction in FS. To calculate AI and FS the following formulae were used:

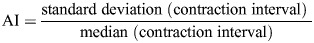


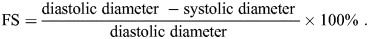


#### Construction of superimposed Poincaré plots and multiple lamin sequence alignment

The variation in the time between contractions was further visualized in a Poincaré plot in which ‘*n*’ describes the time between contractions and is plotted against the time between the subsequent contractions described as ‘*n*+1’. A 0.99 confidence interval ellipse was drawn to visualize the distribution of data points within the graph, and to allow the plotting of multiple Poincaré plots within one graph for easier comparison. Confidence intervals were calculated and plotted with R studio (version 4.3.3). Alignment of lamin protein sequences between species was performed using ClustalO with Jalview (2.11.3.2) (Waterhouse et al., 2009).

### Western blot analyses

After the contractile function measurements, prepupae were collected and frozen at −70°C. Next, prepupae were dissolved in radioimmunoprecipitation assay (RIPA) buffer [2 mM Tris-HCl (PanReac, A1087), 15 mM NaCl (Supelco, 106404), 0.8 µM IGEPAL CA-630 (Sigma-Aldrich, I3021), 12 mM sodium deoxycholate (Sigma-Aldrich, D6750), 1% sodium dodecyl sulfate (SDS; Sigma-Aldrich, 75746)] and subsequently ground using an IKA RW 20 digital at 2000 RPM and homogenized with a sonifier (Branson, SFX 150) continuously for 15 s at 25% amplitude. Then, supernatant was collected after spinning samples down at 24,400 ***g***. Samples were heated at 95°C for 5 min, and equal amounts of protein homogenates were separated by SDS-PAGE (Bio-Rad, 5671084) and transferred onto Immobilon^®^-FL PVDF membrane (Merck, IPFL00010). The amount of transferred protein was checked with a total protein stain (LI-COR, 926-11021) and blocked for 1 h at room temperature with Odyssey^®^ TBS blocking buffer (LI-COR, 927-50000). The membrane was probed with antibodies against α-tubulin (1:5000; Sigma-Aldrich, T9026), acetylated-α-tubulin (1:5000; Sigma-Aldrich, T7451) and PARylation (1:000; Cell Signaling Technology, 83732S) overnight at 4°C. Membranes were subsequently incubated with IRDye 680RD GαM or IRDye800CW GαR (LI-COR). Fluorescent signals were detected with a LI-COR Odyssey Fc imager and quantified using Fiji (v1.52p).

### Statistical analysis

Graphs were constructed using GraphPad Prism (version 9.5.1) and R studio (version 4.3.3). Statistical analyses were performed using R studio (version 4.3.3). R code used to generate figures and statistics, as well as a FIJI macro used in the study, can be found in Dataset 1. *P*<0.05 was considered statistically significant, and error bars represent s.e.m.

## Supplementary Material

10.1242/dmm.052424_sup1 Supplementary information
