# Supplementary material for: Lamin variants cause cardiac arrhythmogenicity in Drosophila
Source: Dis Model Mech. 2025 Jul 25;18(7):dmm052424. doi: 10.1242/dmm.052424 (PMC12320974; doi:10.1242/dmm.052424)
Supplement: Supplementary information [file dmm-18-052424-s1.pdf]

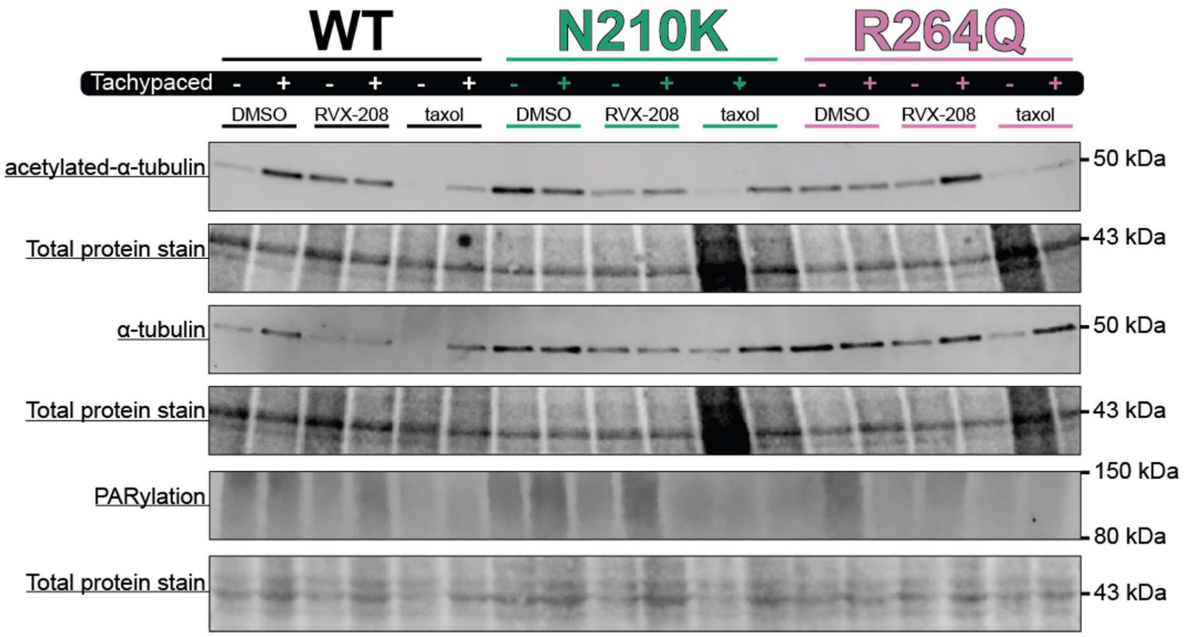

Fig. S1. Western blot analysis with total protein stain reference
Representative Western blot analyses of whole *Drosophila* prepupal lysates as shown in Fig. 4 with corresponding total protein stain (TPS) loading control. The Western blot membranes were stained with a reversible TPS prior to blocking and antibody incubation to assess the total protein load. TPS-stained membranes correspond to the antibody-stained membrane directly above. Membranes were stained for acetylated-α-tubulin, α-tubulin and poly(ADP-ribosyl)ation (PARylation) in prepupae treated with either DMSO, RVX-208 or taxol before tachypacing (BTP) and after tachypacing (ATP). N=3.

Dataset 1. R code and FIJI macro used in the study.

Available for download at
https://journals.biologists.com/dmm/article-lookup/doi/10.1242/dmm.052424#supplementary-data

Disease Models & Mechanisms • Supplementary information
